# Genetic Variant of Glucocorticoid Receptor Gene at rs41423247 and Its Association with Major Depressive Disorder: A Case-Control Study

**DOI:** 10.22086/gmj.v0i0.1181

**Published:** 2018-12-31

**Authors:** Negar Firouzabadi, Hasti Nouraei, Ali Mandegary

**Affiliations:** ^1^Department of Pharmacology & Toxicology, School of Pharmacy, Shiraz University of Medical Sciences, Shiraz, Iran; ^2^Department of Toxicology & Pharmacology, School of Pharmacy, Kerman University of Medical Sciences, Kerman, Iran; ^3^Pharmaceutics Research Center, Neuropharmacology Institute, Kerman University of Medical Sciences, Kerman, Iran

**Keywords:** Glucocorticoid receptor, Genetic variant, Single nucleotide polymorphism, Major, depressive disorder

## Abstract

**Background:**

Extensive distribution of glucocorticoid receptors (GCRs) in different brain areas along with disruption of hypothalamic-pituitary-adrenal (HPA) axis in major depressive disorder (MDD) and the cross talk between GCRs and HPA proposes genetic variants of GC receptor genes as potential contributors in MDD. Among the GCR polymorphisms, rs41423247, rs6195 and rs6189/rs6190 are suggested to be involved in MDD.

**Materials and Methods:**

We investigated the association between rs41423247, rs6195 and rs6189/rs6190 and MDD in a case-control study. One hundred MDD patients along with 100 healthy individuals were enrolled in this study. genetic variants of rs41423247, rs6195 and rs6189/rs6190 were determined in extracted DNAs using PCR-RFLP.

**Result:**

The prevalence of heterozygote and mutant carriers of rs41423247 were significantly and by 1.9 fold greater in cases versus controls (P=0.033; OR; 95%CI=1.9; 1.1-3.3). Moreover, carriers of the mutant (G) allele were by 1.8 fold more prevalent in MDD group (P=0.013; OR;95%CI=1.8; 1.1-2.8).

**Conclusion:**

Specific carriers of rs41423247 might be more susceptible to developing MDD. This supports the hypothesis of the involvement of GCRs in pathophysiology of MDD.

## Introduction

Major depressive disorder (MDD) as an enigmatic psychological disorder with a high prevalence [1], is currently considered as one of the main five causes of disability globally and will be the second foremost cause of disability by the year 2020 [2,3]. Multiple biological and environmental parameters along with genetic and epigenetic factors have been proposed to be linked to MDD [4-6].

However, the exact mechanism involved in pathophysiology of MDD is not clear to date [1]. Glucocorticoids (GCs) having an essential role in controlling neuroendocrine and inﬂammatory and responses to various challenges like stress [7,8], suppress critical inflammatory pathways such as outﬂows of the hypothalamic-pituitary-adrenal (HPA) axis [9]. Malfunction of the GC system in suppressing inflammation results in many illnesses including MDD [10] in which one of the main ﬁndings is the alteration in GC resistance [11,12]. Dysregulation of the HPA axis is proposed to be the foremost cause of depression [13-15]. Forebrain GC receptors (GCRs) have been implicated to contribute to HPA axis regulation and depression. These receptors are extensively found in cognitive regions of the forebrain [16,17]. Decreased GCR mRNA expression is detected in cortex and hippocampus of MDD patients [18] which is surprisingly increased after antidepressant treatment [19,20] and partial GCR resistance is restored [21]. Considering that the exact mechanism of GC resistance in MDD is unknown to date, the influence of genetics in both MDD and GC resistance is undeniable. Therefore genetic factors leading to alteration in GCR sensitivity might affect susceptibility to MDD. It is claimed that some GCR genetic variants can alter the structure of hippocampus and the integrity of its subdivisions which may contribute to MDD [22,23].rs41423247 (BclI) polymorphism is located in intron 2 of GCR gene resulting in displacement of G to C nucleotide [24]. rs6195 (N363S) polymorphism located in exon 2 of GCR gene results in a change of asparagine to serine [25]. These two variants are reported to be associated with GC sensitivity. Another variant located in exon 2 of GCR gene, is rs6189/rs6190 (ER22/23EK) with two linked nucleotide variations in codons 22 and 23 resulting in GCR resistance [26]. In this study we investigated the potential association of three genetic variants of GCRs (rs41423247, rs6189/rs6190 and rs6195) with MDD, for the first time, in a group of depressed Iranian patients.

## Materials and Methods

One hundred MDD patients (male: 33, Female: 67, mean age± SD= 32.58±10.65) were included in our case-control study. They were recruited from Hafez Hospital, Shiraz, Iran. All participants were interviewed by the samepsychiatrist and MDD was diagnosed and confirmed according to Diagnostic and Statistical Manual of Mental Disorders, 5th Edition (DSM-V) criteria [27] and the severity of depression was evaluated with an initial 21 item Hamilton Depression Rating Scale (HDRS) [28].Only patients with no history of antidepressant medication use and negative previous diagnosis of depression were included in our study.patients with the following conditions were excluded from the study: a family history of schizophrenia, a personal history of bipolar disorder, a family history of bipolar disorder in first degree relatives; a personal history of schizophrenia, manic or hypomanic episode, mood incongruent psychotic symptoms, active substance dependence, and current treatment with antipsychotics or mood stabilizers and significant medical condition,inflammatory illnesses such as irritable bowel disease (IBD), rheumatoid arthritis (RA) and gout,cancer, cardiovascular diseases and diabetes.

the control group comprised of 100 healthy and unrelated individuals (male: 44, Female: 56, mean agent± SD: 32.83±13.53) attending routine health checkups who volunteered to be interviewed by the psychiatrist. Healthy volunteers were with no evidence of underlying diseases (psychiatric disorders, auto immune diseases, cancer, IBD, diabetes, hypertension, hyperlipidemia and obesity). all participants were interviewed by the same psychiatrist using a similar protocol (DSM-IV criteria) in order to rule out psychiatric illnesses.

All 200 participants were Iranian, from the same geographical area and born from Iranian parents.

### DNA extraction and genotyping

DNAs of both control and case group were extracted using whole blood and by a standard protocol [29]. PCR amplification of rs41423247, rs6195 and rs6189/rs6190 was performedusing primers and enzymes listed in Table-2.The PCR amplification of variants was performed as previously described [30]. After 3-16 hours of incubation, for separating digested fragments electrophoresis on agarose (Invitrogen Ultra Pure®) gel 2% was performed After staining with ethidium bromide the gel was visualized in a UV trans illuminator. All the samples were double checked and reconfirmed(Table-1).

**Table 1:** Primer Sequences, Restriction Enzymes and Locations of rs41423247, rs6195, and rs6189/rs6190 Polymorphisms on DNA.

**Polymorphisms**	**Primer sequence (5′-3′)**	**DNA bp**	**Restriction enzyme digestion**	**DNA Fragment size(base pair)**	**References**
rs41423247 (BclI)	F-5_TGCTGCCTTATTTGTAAATTCGT -3 R-5_AAGCTTAACAATTTTGGCCATC -3	335	BclI	335/222/117	Bachmann, *et al*. [45] 2005
rs6195 (N363S)	F-5_AGTACCTCTGGAGGACAGAT_3 R-5_GTCCATTCTTAAGAAACAGG_3	243	Tsp509I	153/134/95/19	Huizenga, *et al*. [25] 1998
rs6189/rs6190 (ER22/23EK)	F-5_TGCATTCGGAGTTAACTAAAAAG_3 R-5_ATCCCAGGTCATTTCCCATC _3	448	MnlI	184/163/149/ 50/49/35	Panek ,*et al*. [46] 2014

**Table 2:** Demographic Characteristics of Depressed Patients and Healthy Individuals.

**Variables**	**Depressed Patients**	**Healthy control**	**P value**
Sex (male/female)	33/67	44/56	0.146
Age (years) Mean ± SD	32.58±10.68	32.83±13.53	0.351
BMI (kg/m^2^) Mean ± SD	24.76 ±0.38	24.53 ±0.43	0.356

**SD:** Standard deviation; **BMI:** Body mass index

### Statistics

SPSS 21.0 for Windows (SPSS Inc., Chicago) was used for data analysis. . Chi-square test was used to asses Hardy–Weinberg equilibrium (HWE) for distribution of genotypes . Continuous variables are shown as mean ± SD. Genotype frequencies are shown in percentage (%). Normal Distribution of continuous variables was tested with the Kolmogorov–Smirnov test. to assess genotype and allelic distribution differences between the two groups Pearson’s chi-square or Fisher’s exact test were applied Odds ratio (OR) and 95% conﬁdence intervals (CI) were reported . P value <0.05 was considered as statistically signiﬁcant.

## Result

Demographic characteristics of healthy controls and patients with MDD are listed in Table-2. Characteristics of healthy and MDD groups were compared regarding variables. No significant differences were observed between the two groups with regard to age, sex and BMI (P=0.351, 0.146, 0.356 respectively). Table-3 demonstrates genotype and allele frequencies of MDD individuals and healthy participants. There was a significant association between CG and GG genotype of rs41423247 and MDD (P=0.033; OR; 95%CI=1.9; 1.1-3.3). In addition, G allele of rs41423247 was also associated with depression (P=0.013; OR; 95%CI=1.8; 1.1-2.8). There was no significant association between genotypes and alleles of rs6195 and rs6189/rs6190 variants and MDD (P value˃0.05).

**Table 3:** Frequencies of Three Polymorphisms of the Glucocorticoid Receptor Gene in Healthy and MDD Patients.

**Variant**	**Healthy participants (N=100)**	**MDD Patients (N=100)**	**PC**	**OR**	**95%CI**
**rs41423247**			0.033	1.9	1.1-3.3
CC	59 (59%)	43 (43%)	
CG	39 (39%)	48 (48%)	
GG	2 (2%)	9 (9%)	
**Alleles**			0.013	1.8	1.1-2.8
C	157 (78.5%)	134 (67%)	
G	43 (21.5%)	66 (33%)	
**rs6195**			1	1.5	0.2-9.2
TT	98 (98 %)	97 (97%)	
AA	2 (2%)	3 (3%)	
**Alleles**			0.75	1.5	0.4-5.4
A	196 (98.0 %)	194 (94.0%)	
T	4 (2.0%)	6 (6.0%)	
**rs6189/rs6190**			1	0.8	0.2-2.8
AA	94 (94%)	95 (95%)	
TT	6 (6%)	5 (5%)	
**Alleles**			0.83	0.8	0.3-1.9
A	188 (94.0%	190 (95.0%)			
T	12 (94%)	10 (5.0%)			

**MDD:** Major depressive disorder; **OR:**Odds ratio; **Cl:**Confidence Interval; **PC:**P Value for Chi-square Test

## Discussion

As far as surveyed, this is the first study examining the possible association of MDD with variants of GCR in a depressed Iranian population. Here we found a significant association between CG and GG genotype of rs41423247 and MDD. Moreover, carriers of the G allele of rs41423247 were also by 1.8 fold more prevalent in MDD group versus controls than carriers of the C allele.MDD, the most common psychiatric disorder has the lifetime prevalence of 8-12% [31]. In addition to the neurotransmitters the endcrine system plays an influential part in the pathophysiology of this disorder [32]. Among the many endocrine features present in MDD is the hyperactivity of HPA axis resulting in high serum levels of cortisol, the most important GC in human [33], which binds selectively to GCRs. The GCRs are believed to be the major receptors in the regulation of response to stress in situations like MDD where GC levels are high [12]. After binding to GCRs, this complex translocates into the nucleus. Thereafter it can either bind to GC responsive elements on DNA or directly to transcription factors which results in upregulation or downregulation of transcription of target genes [34]. Studies on psychiatric disorders revealed that GCRs are either deleted or overexpressed in the entire brain, too early in life, therefore it is difficult to attribute particular regions of the brain to each observed phenotype. However, the GCR in the forebrain has been shown to regulate the HPA axis and behaviour under stressed conditions such as MDD [35]. The high expression of GCR in formation of the hippocampus explains the atrophic response to stress and stress hormones in these parts of the human brain [36]. One of the major possibilities regarding the mechanism of GCRs in depression is the primary change in GCR genetic structure [37]. Among the genetic variants of GCRs rs41423247, rs6189/rs6190 and rs6195 were found to be associated with alteration in changes in cortisol levels or GC sensitivity [24,38]. As previously reported, sensitivity and resistance to GCs can both lead to MDD. Heterozygote and mutant variants of rs41423247 are associated with increased sensitivity to GCs and a positive feedback on CRH in the limbic system [24]. However, this variant has a dual tissue-specific characteristic with both increase and decrease sensitivity to GCs. Elevated CRH in the limbic regions has been associated with depressive behaviour [11]. On the other hand increased GCR resistance has been shown to be a hallmark of endocrine dysregulation in MDD [11,12]. In line with the previous study by van Rossum *et al*. our results indicate a positive association between mutant and heterozygote genotypes as well as mutant allele of rs41423247 with development of MDD [38]. A report in polish population also indicated an association between this variant and MDD [39]. Likewise a study in postmenopausal women with depression showed a positive association of rs41423247 with MDD [40]. Confirming our observed results, a report is suggestive of reduction in HDRS core in carriers of mutant variants of rs41423247 [41]. Studies regarding association of rs41423247 variants and response to antidepressants were also in line with our finding [30,38,42,43]. In contrast to our finding a study suggests an association between heterozygote variants of rs41423247 and less vulnerability for recurrent depressive symptoms [44]. Considering the intronic position of rs41423247 and the unknown molecular consequence of this polymorphism on GCR physiology, it is assumed that this variant may be linked to other functional variants in the 3’ untranslated region or the promoter region of the GR gene [41]. Regarding rs6189/rs6190 and rs6195 polymorphisms, our results showed lack of association with MDD. A report in Dutch population however showed a positive association of the mutant form of rs6189/rs6190 polymorphism with MDD and antidepressant treatment but lack of association with regard to rs6195 [38]. Confirming our results, a previous report in an Iranian depressed population is suggestive of lack of association between these variants and response to fluoxetine [30]. These conflicting results might be due to ethnical differences on one hand and the low frequencies of these two rare genetic variants in various populations. In future studies these finding are urged to be replicated in larger populations and with different ethnicities.It is worth mentioning that in this study only the newly diagnosed MDD patients were enrolled, which refers to the absence of other concomitant psychiatric illnesses. Moreover patients with illnesses such as IBD, RA, neoplasms, cardiovascular diseases, gout and diabetes were excluded. Therefore, the impact of other confounding factors are minimized.

## Conflict of interest

The authors declare no conflict of interest.
